# Comparative Evaluation of Mineral Trioxide Aggregate Obturation Using Four Different Techniques—A Laboratory Study

**DOI:** 10.3390/ma14113126

**Published:** 2021-06-07

**Authors:** Abhishek Isaac Mathew, Silvia Chamin Lee, Giampiero Rossi-Fedele, George Bogen, Venkateshbabu Nagendrababu, William Nguyen Ha

**Affiliations:** 1 Adelaide Dental School, University of Adelaide, Adelaide, SA 5000, Australia; abhishek.isaac@gmail.com (A.I.M.); svcmlee@gmail.com (S.C.L.); giampiero.rossi-fedele@adelaide.edu.au (G.R.-F.); 2 School of Dentistry, University of Queensland, Brisbane, QLD 4072, Australia; g.bogen@uq.edu.au; 3 Department of Preventive and Restorative Dentistry, College of Dental Medicine, University of Sharjah, Sharjah 27272, United Arab Emirates; hivenkateshbabu@yahoo.com

**Keywords:** dental cements, dental materials, endodontics, MTA, root canal obturation, root canal therapy

## Abstract

This study aimed to compare the density of mineral trioxide aggregate (MTA) as a root canal filling material in the apical 5 mm of artificial root canals. Forty transparent acrylic blocks with 30-degree curved canals were instrumented and allocated into four compaction technique groups (*n* = 10): Lawaty (hand files); gutta-percha (GP) points; auger (nickel–titanium rotary files in reverse mode); and plugger technique. Filled canals were weighed after setting the MTA to calculate difference in mass. Two postoperative radiographs compared radiopacity by measuring luminance variations at 0.5 mm, 1 mm, 2 mm, 3 mm, 4 mm, and 5 mm from the root apex. Obturation time was measured using a digital chronometer. The significance level was set to *p* < 0.05. The plugger group had a lower mass. Relative luminance was significantly higher for the Lawaty group than the plugger group at all examined apical levels. The relative luminance of the auger and GP groups were significantly higher than the plugger group at depths between 0.5 mm and 2 mm. Relative luminance was highest for the Lawaty technique at all depths between 0.5 mm and 4 mm. The Lawaty technique group was associated with increased obturation time compared with pluggers. Compacting MTA in curved canals with the Lawaty technique has the highest mass and radiopacity but requires more time.

## 1. Introduction

Mineral trioxide aggregate (MTA) is a tricalcium silicate-based cement (TSC) used extensively for various endodontic procedures to repair tissues of the periodontium and pulp [1,2]. MTA’s major constituents are tricalcium silicate, tricalcium aluminate, dicalcium silicate, tetracalcium aluminoferrite and bismuth oxide [2]. The constituents are similar to Portland cement, with the exception of bismuth oxide [2]. Placement of the material can resemble partial root canal obturation radiographically when applied as a root-end filling or during resorption or perforation repair [1]. Its success in these applications is primarily due to its favourable bioactive and physicochemical properties [2]. Moreover, MTA as an orthograde root canal filling material can be an alternative treatment strategy for a contingency of challenging and complex cases [3,4]. Although there are currently many varieties of TSCs available, MTA remains the most broadly used and investigated.

The advantages of using MTA as a root canal obturation material include superior sealing ability [5], improved fracture resistance, repair and regeneration of supporting tissues, and resolution of resorptive defects [6,7,8,9,10]. The cement is effective in treating dental anomalies and anatomical variants [4,11,12,13,14]. Orthograde MTA root canal filling can also be a strategy prior to surgical root-end resection, particularly with limited anatomical access, and can often reduce the indication for surgical intervention [4,15]. After cement hardening, MTA becomes a highly insoluble material that can reduce coronal microleakage, endodontic pathogens and associated biofilms [16,17,18]. However, the removal of the cured cement after canal obturation is extremely difficult in curved canals [19]. Therefore, nonhealing in refractory cases after MTA obturation should be considered for surgical endodontics.

The manipulation of mixed MTA can be challenging in confined anatomical sites, particularly the apical area where voids are commonly generated [11,20,21]. The presence of voids and gaps between the filling material and root canal walls can negatively impact the sealing ability of MTA [11,20]. Additionally, there are no instruments specifically designed for the orthograde obturation of mature teeth, with the exception of OrthoMTA (BioMTA, Seoul, Korea) [22]. Several techniques have been recommended for MTA canal obturation, including lentulo spirals [23], manual compaction using hand files and pluggers [3], paper points or modified GP carriers [24], ultrasonic activation [25,26], and the auger technique (engine-driven nickel–titanium (NiTi) files) [4].

Ultrasonic activation has been directly applied to MTA by placing ultrasonic tips onto the MTA or indirectly applied via placing ultrasonics onto pluggers to achieve greater density [25,26]. However, some of these techniques require specialized MTA carrier guns [27] and pluggers, which may not adequately reach the apical third of the root canal [22]. Therefore, a more reliable orthograde approach for MTA obturation would require instruments that accurately conform to the prepared canal shape to assist in the transfer and compaction of the cement apically [22]. Another important consideration is the clinical time required to complete MTA obturation, particularly in multirooted teeth. To date, no consensus exists on the ideal technique or instruments required for MTA canal obturation, as the current literature shows conflicting results [11,20,25,26].

This investigation compared the mass of MTA as a root canal filling material in the apical 5 mm of artificial root canals by measuring the mass, radiopacity, and obturation time using four different techniques, including the Lawaty technique (hand files), auger technique, gutta-percha (GP) points, and NiTi pluggers. In this study, the null hypothesis was that there is no significant difference in the fill mass, radiopacity, and obturation time when comparing the use of pluggers with the three alternative techniques for the orthograde obturation of root canals using MTA.

## 2. Materials and Methods

### 2.1. Preparation of Samples

An initial working length of 14 mm was established 1 mm short of the apical foramen in 40 transparent acrylic blocks with 30-degree curved canals (Dentsply Maillefer, Ballaigues, Switzerland). The canal length was confirmed by advancing a #10 stainless steel (SS) K-flex file (KavoKerr, Brea, CA, USA) 15 mm into each canal until visible at the apical foramen with 1.0 mm subtracted to preserve the artificial “apical constriction”. Each canal was then instrumented using ProTaper Gold^®^ NiTi rotary files (Dentsply Maillefer) to an ISO 25 apical size with an 8% variable taper. A #10 K-flex file was used to remove the debris and maintain patency by passing the file to the canal foramen (15 mm) after each rotary file used. The canal was irrigated with 1 mL of distilled water between each instrument preparation using a side-vented 27 Gauge needle (Monoject, Tyco Healthcare, Mettawa, IL, USA) 2 mm short of the working length and then dried with paper points (Dentsply Maillefer).

Two preoperative radiographs for each acrylic block were exposed to confirm the absence of any intracanal radiopaque materials before obturation and served as negative controls. Radiographic images were obtained using the Planmeca Intra X-ray machine (Planmeca Oy, Helsinki, Finland) on phosphor plates and scanned using the Kavo Scan eXam One scanner (KavoKerr). Each radiographic film was consistently oriented to the acrylic block using a standardized holder mounted to the tooth block with the central beam directed at a 90° angle to the block/phosphor plate set-up. Each instrumented block was weighed to the nearest 0.01 mg using a Mettler Toledo XSR105DU analytical digital electronic balance (Mettler-Toledo GmbH, Greifensee, Switzerland). The samples were randomly allocated into 4 groups using a computer algorithm program (https://www.random.org, accessed 26 July 2020) by a research assistant not involved in the obturation procedures.

**Figure 1:**
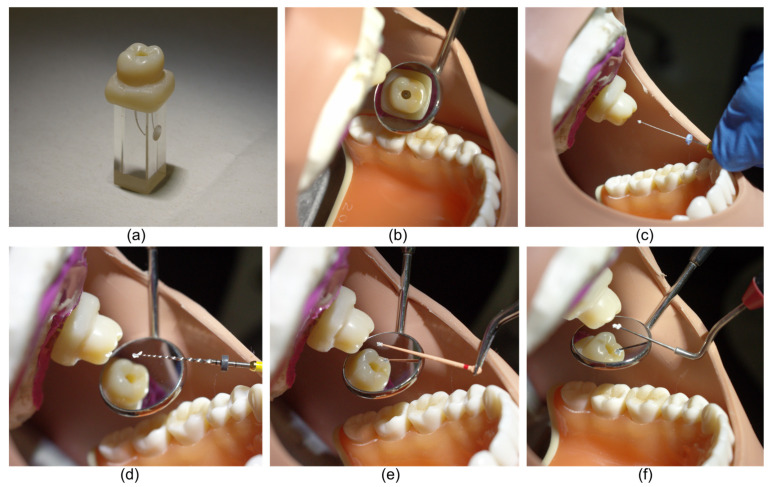
Experimental design: (**a**) acrylic block used with a custom-made composite resin crown; (**b**) acrylic block model after placement in the maxillary tooth mould of the phantom head; (**c**) MTA delivered to the canal using a hand file; (**d**) MTA placed into the canal using a rotary file (ProTaper Gold^®^ F2 not used in image); (**e**) MTA canal delivery using a GP point; and (**f**) MTA transferred to the canal using a plugger.

Plastic maxillary-arch trays mounted on phantom heads were used to standardize each procedure to closely approximate the clinical scenario. The acrylic block was secured within the trays in the maxillary left first molar location using a mold created with 3M Imprint^TM^ 4 Light polyvinyl siloxane impression material (3M, St. Paul, MN, USA). A standardized crown with an access cavity fabricated from Filtek Supreme XTE Universal Dental Composite (3M) was seated over each block, as illustrated in Figure 1.

### 2.2. Experimental Groups

#### 2.2.1. Lawaty Group

**Figure 2:**
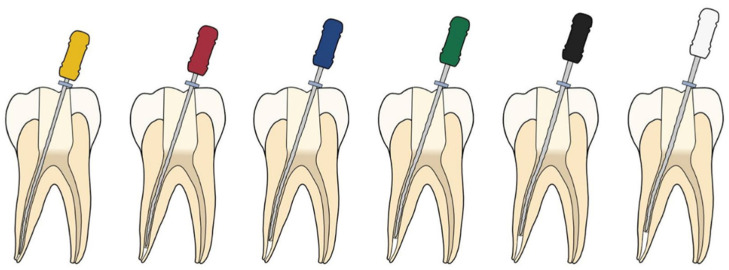
The Lawaty technique: hand files used to carry and pack MTA sequentially in a “step-back” method.

Hand files were used to carry and pack white MTA Angelus (Angelus Solucões Odontológicas, Londrina, Brazil) into the canal using a “step-back” method as seen in Figure 2 [3]. A SS K-flex file one size smaller than the master apical file (MAF), ISO size 20 with 2% taper, was used repeatedly to carry and pack MTA to the prepared length until the file was short of the working length by 1 mm. Subsequently, the next size SS K-flex file, ISO size 25, was used to repeat this step until the MAF was 2 mm short of the working length. The “step back” approach using incrementally larger files was continued until a size of 45 K-flex file completed the compaction 5 mm short of the working length.

#### 2.2.2. Auger Group

MTA compaction was initiated using engine-driven NiTi rotary files in reverse mode, one smaller than the MAF size. A ProTaper Gold^®^ NiTi rotary file of F1 taper (ISO Size 20, 7% taper) carried MTA into the canal and compaction engaged at the working length [4]. This was continued incrementally until 5 mm of MTA was compacted apically. A custom sequence was executed with a KavoKerr elements^TM^ e-motion endodontic motor (KavoKerr) using a 3:1 instrument transmission ratio, torque control mode, anti-clockwise direction of motor rotation, 1.50 N cm torque, and a speed of 300 rpm.

#### 2.2.3. GP Group

A ProTaper Gold^®^ GP point with a matching F2 taper (Dentsply Maillefer) was used to carry and condense MTA similarly to the Lawaty and auger group, starting at working length. The GP point length was initially shortened at 1 mm from the apical tip using surgical scissors and shortened 1 mm progressively after MTA condensation to maintain the matching size/taper until 5 mm was compacted apically. The protocol varied from the technique originally described [28].

#### 2.2.4. Plugger Group

NiTi pluggers were used to transfer and incrementally condense the MTA. A size of 0 (yellow) Buchanan^TM^ plugger (KavoKerr) was advanced apically in a circumferential motion until 5 mm of the cement was compacted apically.

### 2.3. Obturation of Canals

All canals were incrementally obturated using the assigned technique. The MTA was mixed according to the manufacturer’s instructions on a glass slab and covered with dampened gauze to prevent dehydration. Each separate mix of MTA was standardized to 140 mg powder and mixed with sterile water for 30 s to obtain homogenous consistency. After the initial 5 mm of MTA was compacted apically, extra moisture on the compacted MTA was absorbed with an ISO size 45 paper point. Subsequently, a size one (red) Buchanan^TM^ Hand Plugger was used to transfer and incrementally pack MTA in the remaining canal space to the coronal orifice level, and final compression was completed with a size one cotton pellet. The crown was then removed, and a plastic ruler was used to scrape any excess and ensure a flush coronal fill. After the block was removed from the arch tray, external surfaces were wiped with 70% isopropyl alcohol to remove surface debris and excess MTA under magnification by a blinded research assistant. The composite crown was also cleaned between each procedure using isopropyl alcohol.

### 2.4. Obturation Time

Obturation time was measured using a digital chronometer. The time was recorded from the first insertion of cement into each canal until the MTA obturation was completed. Each obturation was complete when the crown was removed from the block.

### 2.5. Evaluation of Canal Fillings

All blocks were numbered and stored individually at room temperature and reweighed 3 h after the completed obturations to accommodate the MTA setting [29]. The MTA mass difference was used as the outcome measure.

Two postoperative radiographs exposed for each block were exported in TIFF format and calibrated using a standardized template on Affinity Designer for Mac version 1.7.2 (Serif Europe, Nottingham, UK). Each radiograph was assessed by comparing radiopacity by measuring luminance within the canals at 0.5 mm, 1 mm, 2 mm, 3 mm, 4 mm, and 5 mm from the apical end. An eyedropper tool with a radius of 10 on the GNU Image Manipulation Program (GIMP) was used to measure the average image whiteness of 20 × 20 pixels at each increment. Luminance was calculated with the average image whiteness measured using an online calculation tool (https://planetcalc.com/7779, accessed 31 July 2020). In order to calibrate the luminance measurements between radiographs, the luminance of the acrylic block was also measured at a standardized location, and the difference between the luminance of each assigned increment and the block was calculated. Relative luminance was calculated as a percentage, and repeated measurements for luminance 2 weeks apart demonstrated an “almost perfect” intraobserver agreement [30].

All outcomes were measured and tabulated by a blinded research assistant and not involved in the procedures. The blinded investigator exposed the pre-and postoperative radiographs, calculated the luminance, measured obturation times, and weighed the blocks preoperatively and postoperatively.

Obturation time and mass differences between groups were assessed using linear regression. Robust standard errors were specified to account for the potential heteroscedasticity. For each outcome, mean values for the plugger group were compared to each of the other groups post hoc. The depth of relative luminance across technique groups was examined using linear mixed-effects modelling, controlling for an observation angle. A quadratic term for depth was included to account for the observed curvilinear relationship between the depth and relative luminance over 0.5 mm to 5 mm. An interaction between the technique group and depth was included to allow for the rate of change in relative luminance to differ between groups. Differences in estimated mean relative luminance between the plugger group and each of the other groups were assessed at depths of 0.5 mm, 1 mm, 2 mm, 3 mm, 4 mm, and 5 mm post hoc. The level of significance was set at *p* < 0.05. Statistical analyses were performed using Stata (Version 15, StataCorp, College Station, TX, USA).

## 3. Results

### 3.1. Obturation Mass

The mean difference in mass (95% CI) in milligrams for the four groups were as follows: Lawaty group 43.78 ± 1.16; auger group 44.49 ± 1.16; GP group 43.73 ± 1.16; plugger group 37.82 ± 1.16. Table 1 shows the marginal effects of the estimated differences in mean obturation mass with the plugger group set as the reference group. Significant differences were found when the plugger group was compared to the other groups (*p* < 0.0001). No significant differences were found among the remaining groups. The mean preoperative mass of the block was 3473.06 mg (SD: 8.19) and the mean postoperative of the blocks were 3515.51 mg (SD: 8.40).

### 3.2. Radiopacity (Relative Luminance)

**Figure 3:**
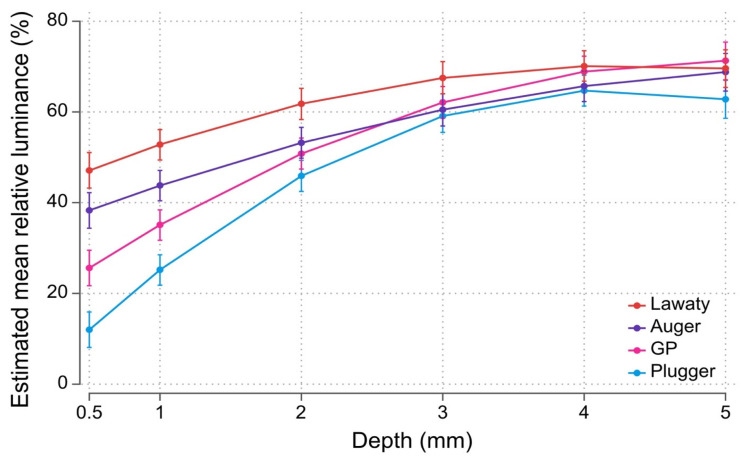
Comparison of the estimated mean values for relative luminance for each group at various depths from the apical end. Estimated differences in mean relative luminance compared to the plugger group generally decreased as the distance from the apex increased.

**Figure 4:**
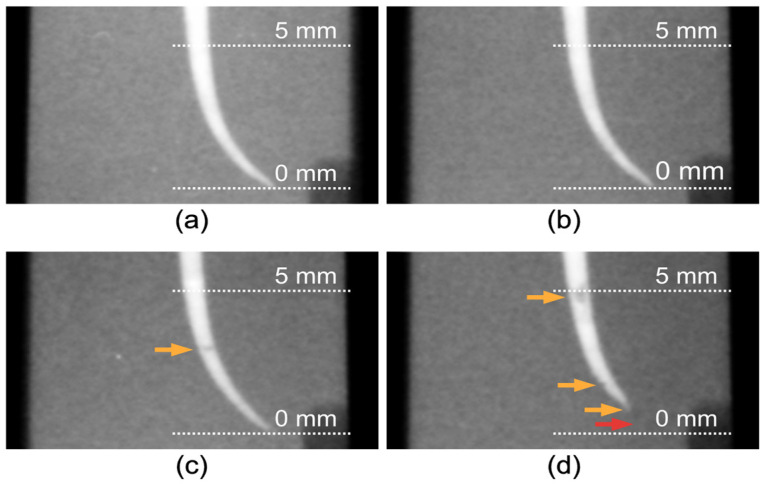
Representative radiographs from each group showing the apical 5 mm: (**a**) Lawaty group; (**b**) auger group; (**c**) GP group; and (**d**) the plugger group. Orange arrows indicate areas of reduced relative luminescence. The red arrow shows that the obturation is short of working length (also an area of reduced relative luminance).

Estimated mean values for each group’s relative luminance at each depth are presented in Figure 3. Table 2 shows the marginal effects of estimated differences in mean relative luminance with the plugger group set as the reference group. Among the four groups, estimated mean values for relative luminance are highest for the Lawaty group at all depths between 0.5 mm and 4 mm from the apical terminus. Estimated mean values for relative luminance at 5 mm are similar among groups. Mean relative luminance was significantly higher for the Lawaty group compared with the plugger group (*p* < 0.0001) at all depths examined. Mean relative luminance for the auger and GP groups were significantly higher (*p* < 0.0001) than the plugger group at depths between 0.5 mm and 2 mm. Within each pairwise comparison, estimated differences in mean relative luminance generally decreased as depth increased in the plugger group. Representative radiographs obtained from each group are shown in Figure 4.

### 3.3. Obturation Time

The mean obturation times (95% CI) in minutes were as follows: Lawaty Group 9.49 (9.19, 9.79); auger 5.67 (5.49, 5.86); GP 5.68 (5.56, 5.80); plugger 5.65 (95% CI 5.60, 5.69). Table 3 shows the marginal effects of the estimated differences in mean obturation times with the plugger group set as the reference group. The Lawaty technique was associated with significantly increased obturation times compared to the NiTi plugger group (*p* < 0.0001). No significant differences were found among the remaining groups.

## 4. Discussion

This investigation suggests that using a plugger for MTA orthograde canal obturation is associated with less fill mass and relative luminance in the apical 4 mm of the root canal when compared with alternative techniques that appear to perform similarly. However, hand file techniques were associated with the longest obturation times. Overall, the null hypothesis was partially rejected. These findings may be related to differences in taper between the apical portion of the artificial canal and the hand file/plugger system. The plugger may have allowed the displacement of MTA in a coronal direction compounded by the challenging task of compacting the cement in a curved canal. Furthermore, despite the NiTi plugger’s flexibility, it may not have provided adequate compaction at the 2 mm level, which represented the point of greatest canal curvature.

The experimental model aimed to closely approximate clinical conditions by placing acrylic blocks in a phantom head maxilla, thus introducing the elements of operator position and intra-oral manipulation of MTA employing different instrument systems. Concurrently, this prevented the direct vision of the canal as the polyvinyl mould and custom composite crown served as blinders, further replicating the clinical setting. The maximum apical file preparation size chosen was F2 (#25), as a #20 K-file for white MTA and #25 K-file for gray MTA has been recommended as the minimum preparation sizes for placing MTA as a root canal filling [4]. The final artificial canal shape selected is compatible with canal dimensions of several root types, although a wide range of anatomical variations occur in the human dentition [31,32].

Two different outcome measures were used to assess obturation quality, including fill mass and relative luminance. Fill mass assumes the MTA is condensed into standardized canal volumes; thus, any increase in mass would correspond to an increase in obturation density [26]. In the present assays, post-obturation mass was recorded after the MTA set time, which was not considered in comparable studies [20,27]. Relative luminance is a novel method to evaluate the radiographic density of digital images based on calculating the average image whiteness (radiopacity) along the filled root canal [33]. By measuring each assigned increment at a standardized location using a template along the block and calculating their difference, any contrast differences between images were corrected.

Based on these findings, the auger technique and matching GP points appear to be the most suitable methods for obturating the apical 5 mm of a curved canal using MTA. Notwithstanding, these obturation findings may not apply to all types of teeth due to wide variations in root canal morphology and, in particular, teeth displaying open apices [34], which is a limitation of the present study. Furthermore, hybrid techniques can be used when required by the clinical scenario. For example, it has been previously reported that it may be challenging to obturate the apical 2–3 mm with the auger technique alone, particularly with abrupt canal curvatures [4]. Therefore, in cases with abrupt apical curvature, the hand file technique may be useful for the apical obturation area and completed using the auger technique [4]. Further studies assessing the various techniques available, alone or combined, in association with other canal morphologies are necessary.

This study utilized a method of replicating the clinical environment as benchtop studies enable easier placement of the MTA which may unrealistically bias the results. This is in contrast to previous studies, as seen in Table 4. Ultrasonics was not used in this study as it would serve as another variable in the study, particularly as there are conflicting studies on whether a difference in the weight and density of the condensed MTA [11,20,25,26].

The limitations of the present study include the use of acrylic blocks with uniform canal volumes without considering the variability of the volume of human dental pulp cavities [35,36,37]. Furthermore, it is assumed that the preparation of the canals would be of equal volume as if they were prepared with identical protocols. Nevertheless, the variation in volume should not have a significant influence on the outcomes as the prepared blocks had blinded and random allocation. Additionally, if natural teeth are used, the anatomical differences in root canal width and dentin thickness may influence the outcomes of luminance values. Thus, acrylic blocks improve standardization. Furthermore, the testing of MTA should often be performed within physiological solution (e.g., phosphate-buffered solution) as some absorption of phosphates is likely to expand and increase the mass of the MTA [29]. However, as this testing was in plastic blocks, there would be minimal transmission of ions compared to if the study was performed in natural teeth that were submerged in physiological solution.

More recent studies have employed micro-CT imaging for volumetric analysis to measure the voids within the root canal [11,20]. Although this study utilized only two-dimensional (2D) imaging to evaluate radiographic density, which may have drawbacks, the 2D radiographic assessment remains a reasonable alternative [38]. At the same time, a densely compacted material based on two-dimensional radiographic assessment improves the treatment outcomes of primary root canal treatment [38]. Finally, the investigation examined only the apical 5 mm of MTA obturation because this apical barrier length has been shown to prevent bacterial leakage [39]. An apical 5 mm of MTA allows post placement when indicated or the backfilling of the canal with thermoplastic GP or resin-based materials [4].

## 5. Conclusions

The Lawaty, auger and matching GP point techniques are comparable techniques in MTA obturation in curved canals. Pluggers are likely to have more voids and be unable to reach the apical end of the canal. Although the Lawaty technique takes the longest time to complete canal obturation, it may produce a greater density obturation with greater radiopacity.

## Figures and Tables

**Table 1:** Results of estimated differences in mean obturation times (95% confidence intervals) with the plugger group set as the reference group.

Placement Technique	Difference in Mean Obturation Mass (mg)	Standard Error	(95% Confidence Interval)		*p*-Value
Lawaty	5.96	0.81	4.31	7.60	<0.01 *
Auger	6.67	0.81	5.03	8.31	<0.01 *
GP	5.90	0.81	4.26	7.54	<0.01 *

Each group (*n* = 10). * indicates statistically significant differences at *p* < 0.05.

**Table 2:** Results of the estimated differences in mean relative luminance (95% confidence intervals) with plugger group set as the reference group.

Position from Apical End (mm)	Placement Technique	Difference in Mean Relative Luminance (%)	Standard Error	(95% Confidence Interval)		*p*-Value
0.5	Lawaty Auger GP	35.10 26.26 13.56	2.83 2.83 2.83	29.55 20.71 8.01	40.64 31.80 19.10	<0.001 * <0.001 * <0.001 *
1	Lawaty Auger GP	27.60 18.57 9.91	2.43 2.43 2.43	22.83 13.80 5.14	32.37 23.34 14.68	<0.001 * <0.001 * <0.001 *
2	Lawaty Auger GP	15.86 7.27 4.93	2.47 2.47 2.47	11.02 2.44 0.09	20.70 12.11 9.77	<0.001 * 0.003 * 0.046 *
3	Lawaty Auger GP	8.47 1.41 3.03	2.55 2.55 2.55	3.48 −3.60 −1.97	13.47 6.41 8.03	0.001 * 0.581 0.235
4	Lawaty Auger GP	5.45 0.97 4.21	2.43 2.43 2.43	0.69 −3.79 −0.55	10.20 5.73 8.96	0.025 * 0.690 0.083
5	Lawaty Auger GP	6.78 5.96 8.47	3.01 3.01 3.01	0.87 0.06 2.56	12.67 11.86 14.37	0.025 * 0.048 0.005

Each group (*n* = 10). * indicates statistically significant differences at *p* < 0.05. Statistical analysis included linear mixed-effects modelling.

**Table 3:** Results of estimated differences in mean obturation times (95% confidence intervals) with the plugger group set as the reference group.

Placement Technique	Difference in Mean Obturation Times (mins)	Standard Error	(95% Confidence Interval)		*p*-Value
Lawaty	3.84	0.15	3.54	4.15	<0.0001 *
Auger	0.03	0.09	−0.16	0.22	0.7835 *
GP	0.03	0.06	−0.10	0.16	0.6551 *

Each group (*n* = 10). * indicates statistically significant differences at *p* < 0.05.

**Table 4:** Comparison of the methodologies and findings of existing studies.

Author (Year)	Tooth Model	Benchtop * vs. Clinical Simulation	Technique Used	Method of Measurement	Finding
Keleş et al. [11]	Moderately curved (15–20 degrees) mesial roots from mandibular molars with a Vertucci type II canal configuration prepared with Reciproc R25 (size 25, variable taper)	Benchtop	Plugger, ultrasonically activated plugger	Micro-CT—percentage of voids	No significant difference between techniques at apical 3 mm; no significant difference between the two techniques at the coronal half of the isthmus
El-Ma’aita et al. [20]	Minimally curved single canal; anterior human teeth with crowns removed prepared with Protaper F5 (size 50, variable taper)	Benchtop	Plugger ultrasonic activated plugger	Micro-CT—percentage of voids	Ultrasonic compaction produced significantly less-dense root fillings
Yeung et al. [26]	Acrylic blocks with 30-degree curved canals and straight canals prepared with K3 (size 45, 6% taper)	Benchtop	Plugger ultrasonic activated plugger	Fill density using difference in mass	Pluggers with US activation resulted in an MTA fill that was statistically significantly heavier
Aminoshariae et al. [25]	Polypropylene tubes with an inner diameter of 0.7 mm at the apical tip	Benchtop	Plugger direct ultrasonic placement	Void scores using radiographs and light microscope	Pluggers resulted in better adaptation to the tube walls and fewer voids
Present study	Acrylic blocks with 30-degree curved canals prepared to F2 (size 25, variable taper); blocks have resin crowns and are mounted in a phantom head	Clinical simulation	Lawaty auger GP plugger	Fill density using difference in mass relative luminance (radiopacity); obturation time	The Lawaty technique has the highest relative luminance and mass but also consumes the most time; the plugger technique has the lowest relative luminance and mass

* Benchtop studies examine procedures in teeth placed on a table or hand-held by the researchers. Clinical simulation models involve teeth positioned in dental training manikins that simulate the challenges of vision and access.

## Data Availability

The data presented in this study are available on request from corresponding author.

## References

[1] Parirokh M., Torabinejad M. (2010). Mineral trioxide aggregate: A comprehensive literature review—Part III: Clinical applications, drawbacks, and mechanism of action. J. Endod..

[2] Parirokh M., Torabinejad M. (2010). Mineral trioxide aggregate: A comprehensive literature review—Part I: Chemical, physical, and antibacterial properties. J. Endod..

[3] Bogen G., Kuttler S. (2009). Mineral trioxide aggregate obturation: A review and case series. J. Endod..

[4] Bogen G., Lawaty I., Chandler N., Torabinejad M. (2014). MTA root canal obturation. Mineral Trioxide Aggregate: Properties and Clinical Applications.

[5] Sarkar N.K., Caicedo R., Ritwik P., Moiseyeva R., Kawashima I. (2005). Physicochemical basis of the biologic properties of mineral trioxide aggregate. J. Endod..

[6] Al-Hezaimi K., Naghshbandi J., Oglesby S., Simon J.H., Rotstein I. (2005). Human saliva penetration of root canals obturated with two types of mineral trioxide aggregate cements. J. Endod..

[7] Hayashi M., Shimizu A., Ebisu S. (2004). MTA for obturation of mandibular central incisors with open apices: Case report. J. Endod..

[8] D’Arcangelo C., D’Amario M. (2007). Use of MTA for orthograde obturation of nonvital teeth with open apices: Report of two cases. Oral Surg. Oral Med. Oral Pathol. Oral Radiol. Endod..

[9] Aslan T., Esim E., Üstün Y., Özkan H.D. (2021). Evaluation of stress distributions in mandibular molar teeth with different iatrogenic root perforations repaired with Biodentine or MTA: A finite element analysis study. J. Endod..

[10] Tsai Y.-L., Ian W.-H., Jeng J.-H. (2006). Treatment of pulp floor and stripping perforation by mineral trioxide aggregate. J. Formos. Med. Assoc..

[11] Keleş A., Torabinejad M., Keskin C., Sah D., Uzun İ., Alçin H. (2017). Micro-CT evaluation of voids using two root filling techniques in the placement of MTA in mesial root canals of Vertucci type II configuration. Clin. Oral Investig..

[12] Woelber J.P., Bruder M., Tennert C., Wrbas K.-T. (2014). Assessment of endodontic treatment of c-shaped root canals. Swiss Dent. J..

[13] Teixidó M., Abella F., Duran-Sindreu F., Moscoso S., Roig M. (2014). The use of cone-beam computed tomography in the preservation of pulp vitality in a maxillary canine with type 3 dens invaginatus and an associated periradicular lesion. J. Endod..

[14] Kfir A., Telishevsky-Strauss Y., Leitner A., Metzger Z. (2012). The diagnosis and conservative treatment of a complex type 3 dens invaginatus using cone beam computed tomography (CBCT) and 3D plastic models. Int. Endod. J..

[15] Habibi M., Ghoddusi J., Habibi A., Mohtasham N. (2011). Healing process following application of set or fresh mineral trioxide aggregate as a root-end filling material. Eur. J. Dent..

[16] Saunders W.P., Saunders E.M. (1994). Coronal leakage as a cause of failure in root-canal therapy: A review. Endod. Dent. Traumatol..

[17] Yoo J.S., Chang S.-W., Oh S.R., Perinpanayagam H., Lim S.-M., Yoo Y.-J., Oh Y.-R., Woo S.-B., Han S.-H., Zhu Q. (2014). Bacterial entombment by intratubular mineralization following orthograde mineral trioxide aggregate obturation: A scanning electron microscopy study. Int. J. Oral Sci..

[18] Alsalleeh F., Chung N., Stephenson L. (2014). Antifungal activity of Endosequence root repair material and mineral trioxide aggregate. J. Endod..

[19] Boutsioukis C., Noula G., Lambrianidis T. (2008). Ex vivo study of the efficiency of two techniques for the removal of mineral trioxide aggregate used as a root canal filling material. J. Endod..

[20] El-Ma’aita A.M., Qualtrough A.J.E., Watts D.C. (2012). A micro-computed tomography evaluation of mineral trioxide aggregate root canal fillings. J. Endod..

[21] Jho W., Park J.W., Kim E., Song M., Seo D.G., Yang D.K., Shin S.J. (2016). Comparison of root canal filling quality by mineral trioxide aggregate and gutta percha cones/AH plus sealer. Dent. Mater. J..

[22] Alsulaimani R.S. (2016). Single-visit endodontic treatment of mature teeth with chronic apical abscesses using mineral trioxide aggregate cement: A randomized clinical trial. BMC Oral Health.

[23] Vizgirda P.J., Liewehr F.R., Patton W.R., McPherson J.C., Buxton T.B. (2004). A comparison of laterally condensed gutta-percha, thermoplasticized gutta-percha, and mineral trioxide aggregate as root canal filling materials. J. Endod..

[24] Giovarruscio M., Uccioli U., Malentacca A., Koller G., Foschi F., Mannocci F. (2013). A technique for placement of apical MTA plugs using modified Thermafil carriers for the filling of canals with wide apices. Int. Endod. J..

[25] Aminoshariae A., Hartwell G.R., Moon P.C. (2003). Placement of mineral trioxide aggregate using two different techniques. J. Endod..

[26] Yeung P., Liewehr F.R., Moon P.C. (2006). A quantitative comparison of the fill density of MTA produced by two placement techniques. J. Endod..

[27] Krell K., Madison S. (1985). The use of the messing gun in placing calcium hydroxide powder. J. Endod..

[28] Chang S.W., Oh T.S., Lee W., Cheung G.S., Kim H.C. (2013). Long-term observation of the mineral trioxide aggregate extrusion into the periapical lesion: A case series. Int. J. Oral Sci..

[29] Ha W.N., Nicholson T., Kahler B., Walsh L.J. (2017). Mineral trioxide aggregate-A review of properties and testing methodologies. Materials.

[30] Landis J.R., Koch G.G. (1977). The measurement of observer agreement for categorical data. Biometrics.

[31] Wu M.K., R’oris A., Barkis D., Wesselink P.R. (2000). Prevalence and extent of long oval canals in the apical third. Oral Surg. Oral Med. Oral Pathol. Oral Radiol. Endod..

[32] Ahmed H.M.A., Rossi-Fedele G. (2020). Preferred reporting items for root and canal anatomy in the human dentition (PROUD 2020)-A systematic review and a proposal for a standardized protocol. Eur. Endod. J..

[33] Duarte M.A.H., de Oliveira El Kadre G.D., Vivan R.R., Guerreiro-Tanomaru J.M., Filho M.T., de Moraes I.G. (2009). Radiopacity of Portland cement associated with different radiopacifying agents. J. Endod..

[34] Hartmann R.C., Fensterseifer M., Peters O.A., De Figueiredo J.A.P., Gomes M.S., Rossi-Fedele G. (2019). Methods for measurement of root canal curvature: A systematic and critical review. Int. Endod. J..

[35] Loizides A., Eliopoulos D., Kontakiotis E. (2006). Root canal transportation with a Ni-Ti rotary file system and stainless steel hand files in simulated root canals. Quintessence Int..

[36] Alodeh M.H., Doller R., Dummer P.M. (1989). Shaping of simulated root canals in resin blocks using the step-back technique with K-files manupulated in a simple in/out filling motion. Int. Endod. J..

[37] Fanibunda K.B. (1986). A method of measuring the volume of human dental pulp cavities. Int. Endod. J..

[38] Ng Y.L., Mann V., Rahbaran S., Lewsey J., Gulabivala K. (2007). Outcome of primary root canal treatment: Systematic review of the literature—Part 2. Influence of clinical factors. Int. Endod. J..

[39] Al-Kahtani A., Shostad S., Schifferle R., Bhambhani S. (2005). In-vitro evaluation of microleakage of an orthograde apical plug of mineral trioxide aggregate in permanent teeth with simulated immature apices. J. Endod..

